# Supplementary material for: Suicidal behaviours and self-harm among adolescents: Results from a school-based mental health survey in the Philippines
Source: Glob Ment Health (Camb). 2025 Dec 2;12:e150. doi: 10.1017/gmh.2025.10105 (PMC12720380; doi:10.1017/gmh.2025.10105)
Supplement: Alberto et al. supplementary material [file S2054425125101052sup001.docx]

**Table S1. Questionnaire modules**

| **Module** | **Content** |
| --- | --- |
| Demographic characteristics | Questions related to gender, age, grade, family structure, perceived economic status of the family, place of birth, main language |
| Aches | Questions related to pain-related somatic symptoms |
| Sleep | An item about the frequency of experiencing sleep difficulties |
| Body image and eating behaviours | Questions about their thoughts about their body, and problematic eating behaviours |
| Socio-emotional wellbeing | Strengths and Difficulties Questionnaire |
| Suicidal behaviours and self-harm | Questions regarding suicidal ideation, suicide attempts and self-harm |
| Social support and environment | Questions regarding formal and informal sources of help accessed in the past six months, peer relationships, school environment, and loneliness. |
| Bullying | Item about the frequency of traditional and cyberbullying victimization and perpetration inside and outside school, and characteristics of the perpetrator. |
| Environmental threats | Questions regarding emotions towards pandemics, war, climate change and natural disasters. |
| COVID-19 | Questions about the impact of the COVID-19 pandemic on emotions, wellbeing and daily life. |
